# Thymoquinone controlled obesity and invigorated cognitive and memory performance in rats consuming a high-fat diet via modulating oxidative stress, inflammation and apoptosis

**DOI:** 10.1038/s41598-025-05716-4

**Published:** 2025-06-20

**Authors:** Mostafa D. Mostafa, Maggie E. Amer, Magda A. ElKomy, Azza I. Othman, Mohamed A. El‑Missiry

**Affiliations:** Department of Zoology, Faculty of Science, Mansoura University, Mansoura, Egypt

**Keywords:** Brain, Antioxidants, Memory, Cognitive function, Obesity, Inflammation, Biological techniques, Cell biology, Physiology, Zoology

## Abstract

The current study investigated the mitigating effects of thymoquinone (TQ) against high-fat diet (HFD)-mediated brain injury, cognitive and memory impairment, and the underlying mechanisms. Twenty-four adult male Wistar rats were divided into four groups of six rats each. Rats were fed HFD for 12 weeks to induce obesity. On the 9th week, TQ was administered orally to obese rats for four weeks. The effects of TQ were estimated by neurobehavioral testing, biochemical analysis, DNA damage, molecular docking, and histopathological examination of brains and visceral fat. TQ reduced body weight, body weight gain and adipocyte size, improved hyperlipidemia, and normalized the levels of leptin and adiponectin. TQ significantly attenuated the increase in HbA1c percent and insulin resistance. TQ decreased the accumulation of amyloid-β and tau proteins and improved the levels of neurotransmitters in the brains of obese rats. TQ-treated obese rats showed improved thickening of the pyramidal cell layer in the hippocampus and improved cognitive function and memory impairments. Molecular docking analysis indicated that TQ exhibited a marked affinity for inhibiting binding sites of tau and amyloid-β proteins. Furthermore, TQ controlled oxidative stress and enhanced the Nrf2 expression in the pyramidal cell layer and the activity of HO-1, SOD, and CAT in the brain. The restoration of redox balance by TQ was associated with normalization of inflammatory indicators and alleviation of DNA damage in the brains of HFD-treated animals. These changes contributed to the normalization of mitochondrial apoptotic pathway mediators (p53, Bcl-2, Bax, and caspase-3) and maintained the histological structure of the hippocampus. In conclusion, TQ attenuated brain injury, cognitive impairment, and memory deficit with improvement of body weight gain and metabolic status in obese rats through interrelated biological processes, including regulation of redox balance, inflammatory response, neurotransmitter equilibrium, and regression of DNA injury and apoptosis.

## Introduction

Overweight and obesity, associated with excessive consumption of high-fat diets^1^, are public health concerns around the world due to the significant adverse risk on all ages and organs, particularly the brain^2,3^. Chronic consumption of a high-fat diet (HFD) is associated with cognitive impairment, accelerated memory loss, and psychiatric behavioral abnormalities^4^. After short-term HFD consumption, mice showed signs of memory impairment and depressive-like behavior^5^. Furthermore, rats exposed to a high-cholesterol diet developed substantial deficiencies in long-term memory and spatial learning^6^.

Despite epidemiological and animal studies indicating that obesity has an effect on brain functions, the effect of HFD consumption on cognitive and memory performance and neuronal biochemical processes is still unclear. Increased oxidative stress and neuroinflammation are important processes involved in brain disorders^7^. It is reported that inflammation contributes to the detrimental pathological effects of HFD-induced obesity, such as behavioral abnormalities, increased cognitive decline, and dementia^8^. It is stated that HFD-induced cognitive and social impairments are due to dysregulating brain redox balance and insulin resistance^9^. Long-term HFD consumption results in disruption of antioxidant levels and development of oxidative stress, neuroinflammation, and cognitive impairment^10^. These findings underline the importance of searching for safe nutritional substances to control the accumulation of fats in the body to maintain brain health.

Herbal therapy has been utilized extensively recently to treat a wide range of neurological illnesses. Phytomedicine have several advantages, including low toxicity, accessibility, and ease of administration^11^. Thymoquinone (TQ) is the main bioactive ingredient in the *Nigella sativa* seeds. Due to its low molecular weight and lipophilic nature, TQ is able to pass biological membranes like the blood–brain barrier and therefore, it is believed to have potential as a nervous system medication^12^.

TQ possesses important biological activity, including antioxidant, antioxidative^13^^,^^14^ and anti-inflammatory properties^15^. Furthermore, TQ showed pharmacological effects such as hepatoprotective, neuroprotective, and nephroprotective^16^. An interesting recent study showed that TQ reduced oxidative stress, the loss of GABAergic neurons, and memory deficit in the hippocampus after cypermethrin exposure by activating the Nrf2/ARE signaling pathway^17^. Studies have demonstrated that TQ protects cultured rat primary neurons from Aβ-induced neurotoxicity and has an acetylcholinesterase inhibiting action^18^. Furthermore, TQ maintains rat cerebellar neurons by reducing oxidative stress and inflammation after supplementing with a HFD^19^. TQ treatment increased the total antioxidant capacity of cerebellar tissues, decreased lipid peroxidation, and improved the contents of protein and saturated and unsaturated lipids^20^. Obesity is clearly established to cause inflammation and oxidative stress. Overproduction of pro-inflammatory cytokines and reactive oxygen species (ROS) has been linked to cognitive decline and brain injury induced by consuming HFD^7^. TQ has an enhancing effect on cell survival and neurogenesis in healthy hippocampus^21^. Consequently, we postulated that TQ would significantly protect the structure and function of the brains of HFD-fed rats and could be a therapeutic approach to enhance their brain functions due to its anti-inflammatory and antioxidant properties. Despite the information on the mitigating effect of TQ against brain injury and cognitive dysfunction HFD consumers are insufficient, research on TQ interventions for oxidative stress and neuroinflammation caused by the HFD is still in its infancy. Although, the precise mechanisms causing obesity-induced brain damage have not fully clarified, the primary molecular mechanisms include increased oxidative stress, neuroinflammation and apoptosis. Given the wealth of information regarding TQ’s potential molecular neuroprotective effects under various conditions, we aimed to investigate the neuromitigating effects of TQ and its precise mechanisms of action against brain injury, cognitive and memory impairments in the brains of rats fed a HFD.

## Results

The obtained results revealed that treatment with TQ alone had insignificant changes in the measured parameters when compared to the control group.

### TQ reduced body weight and adipocyte size

**Fig. 1:**
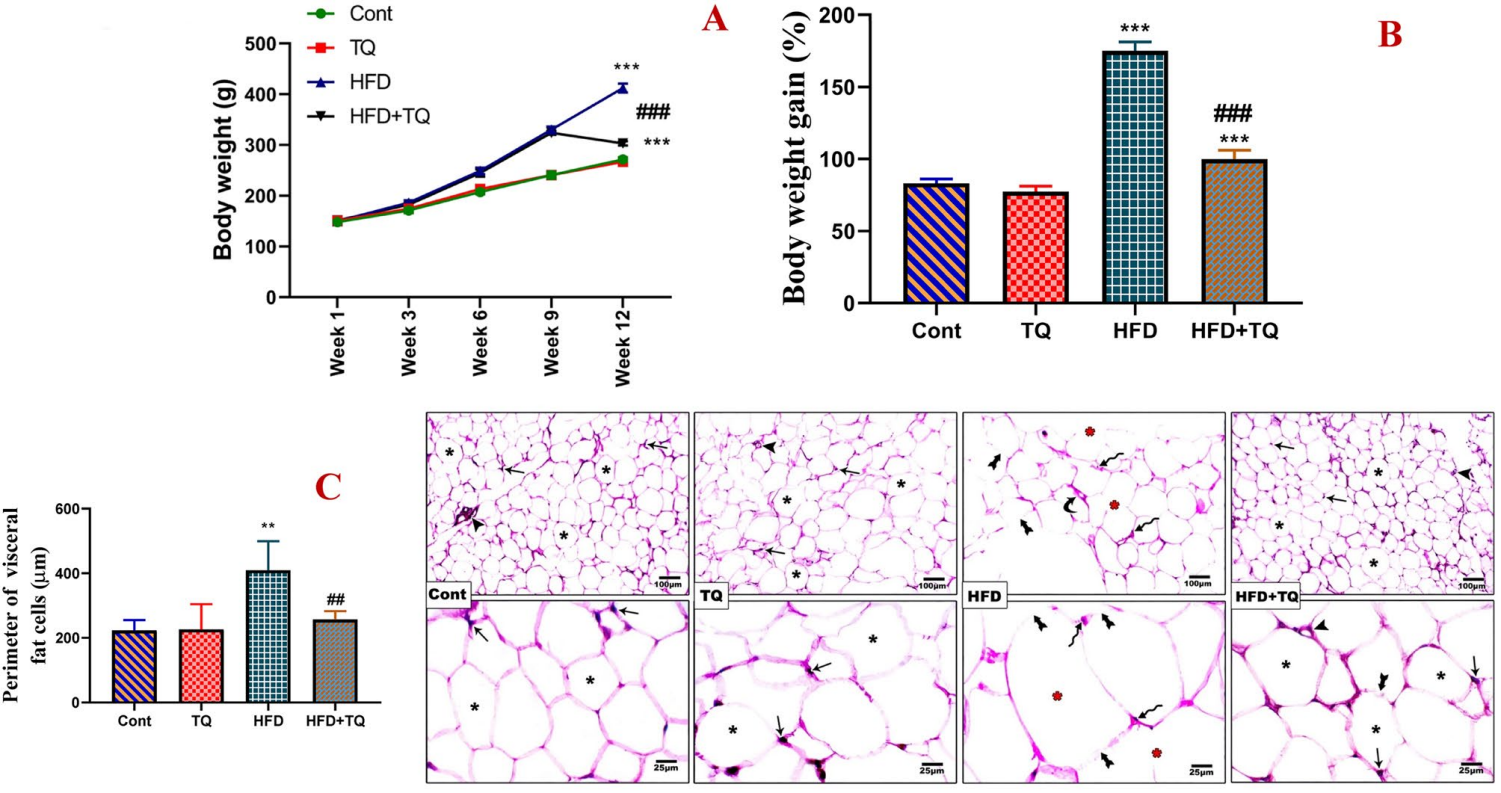
Effect of TQ and HFD on body weight changes (**A**) and body weight gain (%) (**B**). Data are shown as mean ± SEM (n = 6). H&E-stained fat tissue sections of control and different treated groups (**C**). Control and TQ-treated rats revealed normal arrangement and appearance of fat cells (**asterisk**) and nuclei (**arrow**) as well as blood vessels (**arrowhead**). Fat tissue section of the HFD group showed irregular appearance of fat cells represented by widened and swollen fat cells (**red asterisk**) with faded and ruptured cell membranes (**tailed arrow**), pyknosis (**zigzag arrow**), and congestion of blood vessels (**curved arrow**). Fat tissue section of the HFD + TQ-treated rats revealed a remarkable amelioration of fat cell structure and appearance compared to the HFD group. Quantification is expressed as the perimeter of visceral fat tissue (µm) in all studied groups. Each value represents the mean ± SEM of 5 microscopic fields/tissue samples.**,^**##**^ Significant at *P* < 0.01, and ***,^**###**^ Significant at *P* < 0.001. **, *** Indicate comparisons with respect to the control group. ^##,###^ Indicate comparisons with respect to the HFD group. Cont : Control, TQ: Thymoquinone, HFD: High-fat diet, HFD + TQ: High-fat diet + Thymoquinone.

When compared to the control group, daily HFD feeding for 12 weeks significantly (P < 0.001) increased body weight and, consequently, body weight gain percentage (175%). However, as compared to the HFD-fed animals, TQ supplementation to the HFD-fed rats from week 9 to week 12 significantly (P < 0.001) reduced body weight and body weight gain percentage (100%) (Fig. 1 A & B).

Histopathological examination of visceral fats of control and TQ-treated rats revealed normal arrangement and appearance of adipocytes and blood vessels in between. In contrast, the fat tissue section of the HFD group showed an irregular appearance of fat cells represented by a significant (P < 0.01) increase and widening in the perimeter of fat cells with faded and distorted cell membranes, deterioration of nuclei, and pyknosis with congestion of blood vessels. On the other hand, the fat tissue section of the HFD + TQ group revealed a significant (P < 0.01) amelioration of the adipocyte’s perimeter and appearance compared to the HFD group (Fig. 1 C).

### TQ improved cognitive function and memory in HFD-fed rats

**Fig. 2:**
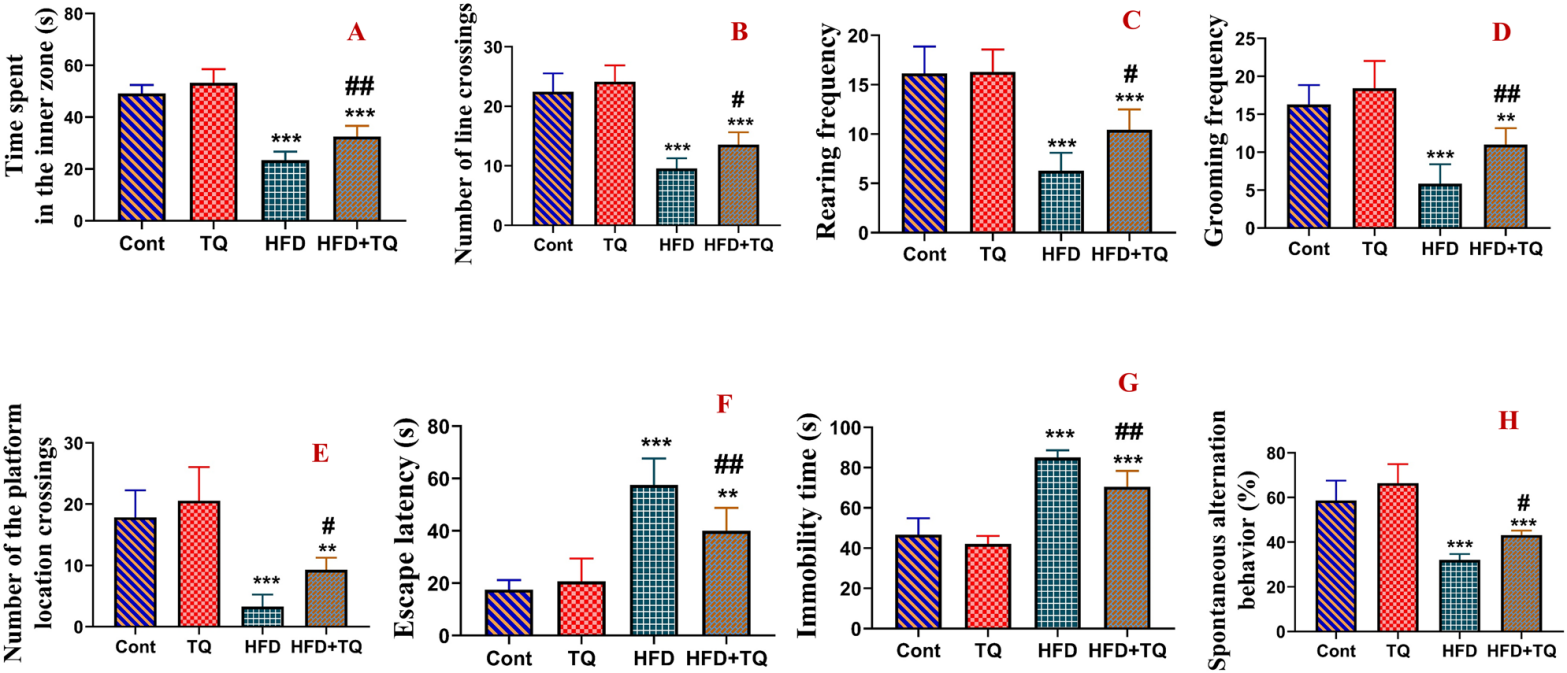
Effect of TQ and HFD on time spent in the inner zone (s; **A**), number of line crossings (**B**), rearing frequency (**C**), grooming frequency (**D**), number of the platform location crossings (**E**), escape latency (s; **F**), immobility time (s; **G**), spontaneous alternation behavior (%; **H**) in different treated groups. Data are shown as mean ± SEM (n = 6).^**#**^ Significant at *P* < 0.05,**,^**##**^ Significant at *P* < 0.01, and***Significant at *P* < 0.001.**,*** indicate comparisons with respect to the control group. ^#,##^ indicate comparisons with respect to the HFD group.Cont: Control, TQ: Thymoquinone, HFD: High-fat diet, HFD + TQ: High-fat diet + Thymoquinone.

The open field test was used to test the effects of HFD and TQ on locomotor, exploratory, and anxiety-like behavior (Fig. 2 A–D). The HFD-treated group showed a significant (P < 0.001) decrease in the time spent in the center area, number of line crossings, rearing, and grooming frequency. On the other hand, TQ administration for four weeks in the HFD treated group significantly (P < 0.01; P < 0.05) improved the time spent in the center squares, grooming frequency, number of line crossings, and rearing frequency.

Depression, short-term spatial memory, and long-term spatial memory were assessed using the Y-maze test and the Morris water maze, and forced swimming (Fig. 2 E–H). Rats fed a HFD required longer (P < 0.001) time to reach the platform and immobility time as well as showed a significant (P < 0.001) decrease in the number of platform location crossings and percentage of spontaneous alternation behavior when compared to the control group. Interestingly, these effects were significantly (P < 0.01; P < 0.05) improved when TQ was administered to HFD-fed rats.

### TQ improved HFD-induced hyperglycemia, insulin resistance, and hyperlipidemia

**Fig. 3:**
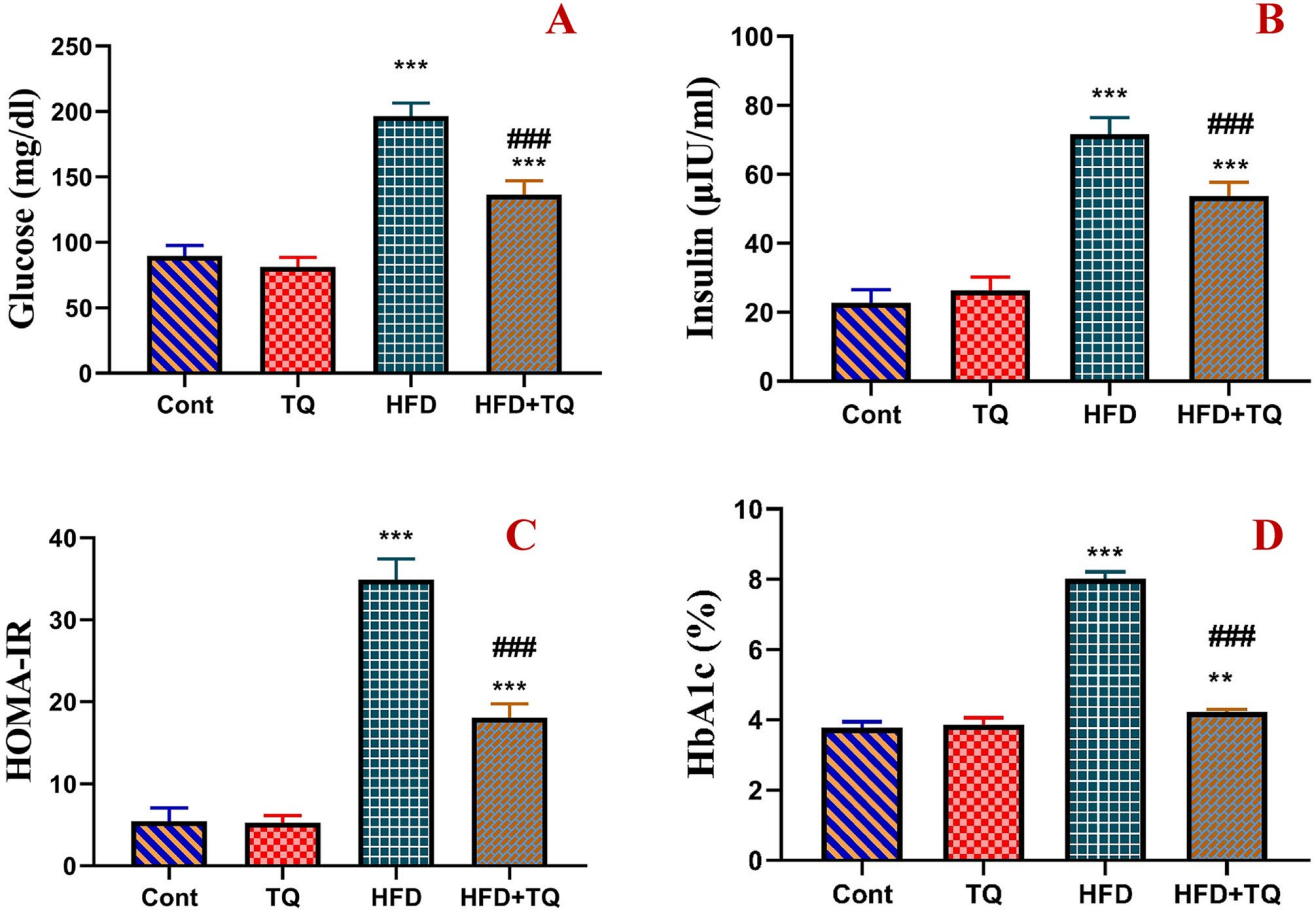
Effect of TQ and HFD on blood glycemic parameters in different experimental groups. Glucose (mg/dl; **A**), insulin (μIU/ml; **B**), insulin resistance HOMA-IR (**C**), and glycosylated hemoglobin A1 c % (HbA1c %; **D**). Data are shown as mean ± SEM (n = 6).** Significant at *P* < 0.01, and ***,^**###**^ Significant at *P* < 0.001.**,*** Indicate comparisons with respect to the control group. ^###^ Indicate comparisons with respect to the HFD group. Cont: Control, TQ: Thymoquinone, HFD: High-fat diet, HFD + TQ: High-fat diet + Thymoquinone.

The levels of insulin, glucose, HOMA-IR, and HbA1c were significantly (P < 0.001) higher than the levels observed in the control rats after 12 weeks of daily HFD feeding (Fig. 3). On the other hand, TQ given to HFD rats for 4 weeks significantly (P < 0.001) improved the glycemic parameters when compared to the HFD group.

**Fig. 4:**
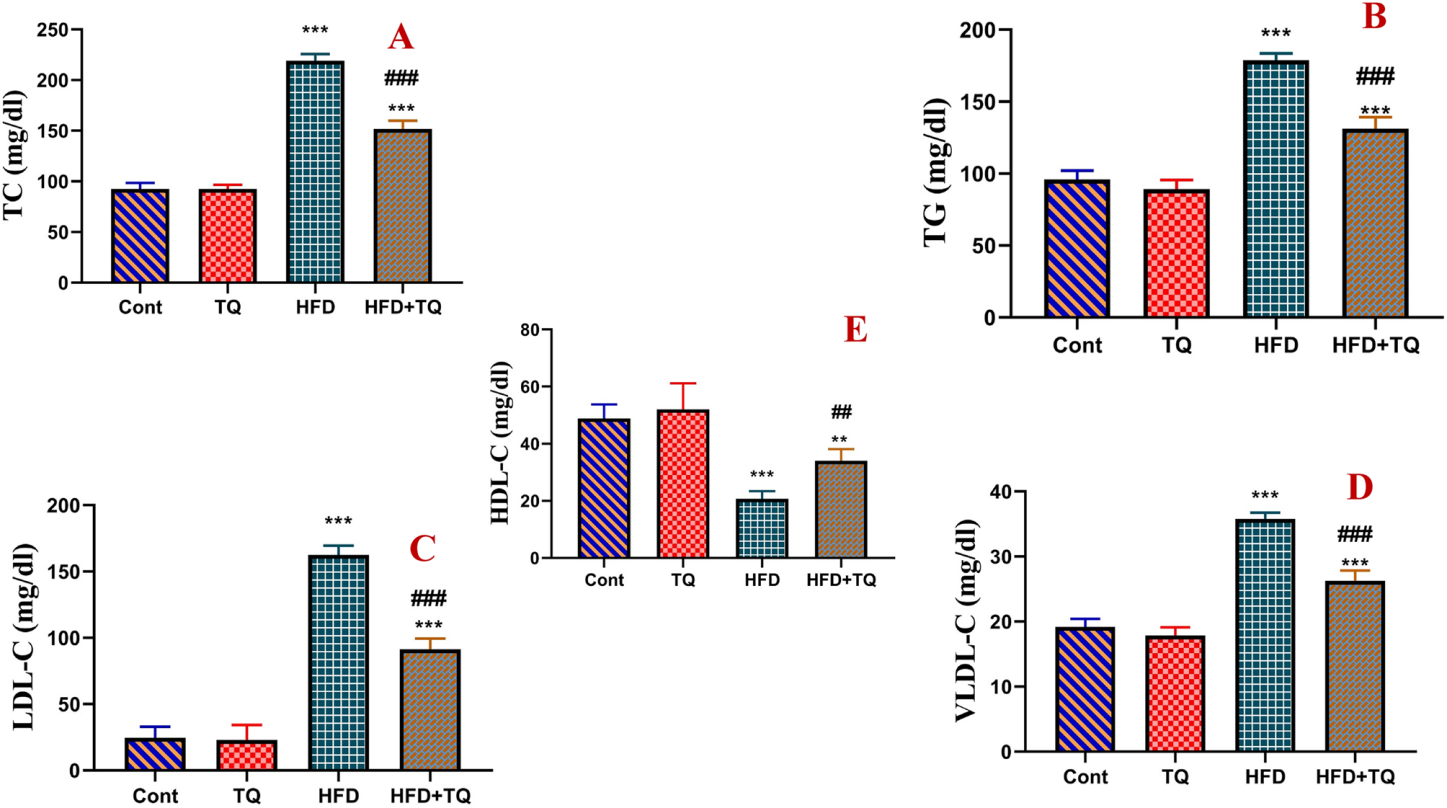
Effect of TQ and HFD on lipid profile in different experimental groups. Total cholesterol (TC; A), Triglycerides (TG; B), low-density lipoprotein (LDL-C; C), very low-density lipoprotein (VLDL-C; D), and high-density lipoprotein (HDL-C; E) are expressed as mg/dl in different treated groups. Data are shown as mean ± SEM (n = 6).**,^**##**^ Significant at *P* < 0.01, and ***,^**###**^ Significant at *P* < 0.001.**,*** Indicate comparisons with respect to the control group. ^##,###^ Indicate comparisons with respect to the HFD group. Cont: Control, TQ: Thymoquinone, HFD: High fat diet, HFD + TQ: High fat diet + Thymoquinone.

Figure 4 displays the effect of TQ and HFD on lipid profiles. After 12 weeks of daily HFD feeding, rats had significantly (P < 0.001) higher levels of TC, TG, LDL-C, and VLDL-C and lower HDL-C levels than the control group. TQ supplementation resulted in a significant (P < 0.001) decrease in TC, TG, LDL-C, and VLDL-C and a significant (P < 0.01) increase in HDL-C levels when compared to the HFD group.

### TQ improved redox balance in HFD-fed rats

**Fig. 5:**
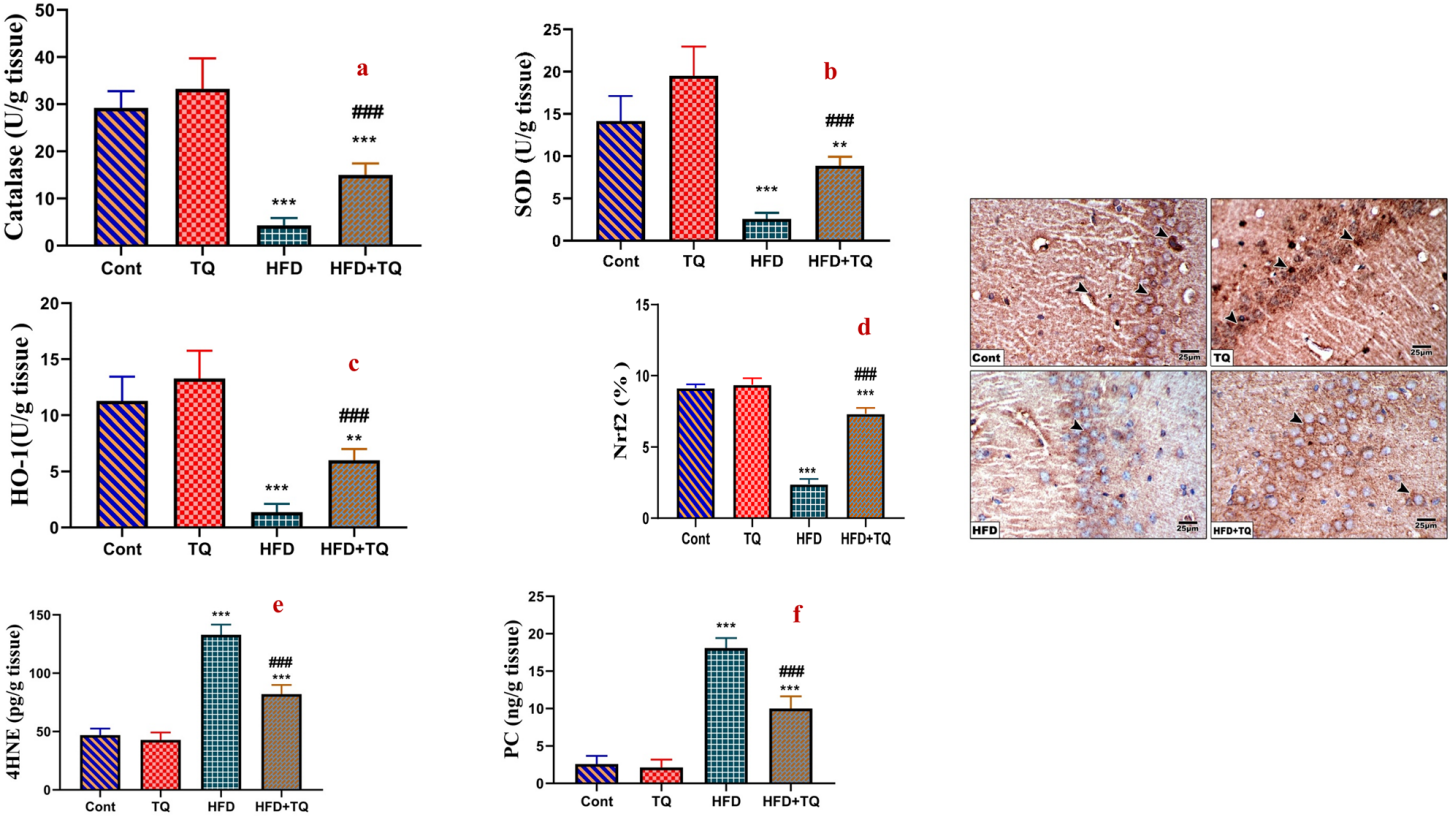
Effect of TQ and HFD on the levels of SOD (U/g tissue) (**A**), catalase (U/g tissue) (**B**), HO-1 (U/g tissue) (**C**), and immunohistochemical expression of Nrf2 in the pyramidal cell layer of hippocampal sections in the control and different rat groups (**D**). Expression is indicated by arrowhead (IHC, 400). Values are expressed as the means ± SEM of 5 microscopic fields/tissue samples of Nrf2%. As well as oxidative stress markers (4HNE, pg/g tissue) (**E**) and protein oxidation (protein carbonyl) (PC, ng/g tissue) (**F**) in the brain of all experimental groups. Data are shown as mean ± SEM (n = 6).**Significant at *P* < 0.01, and ***, ^**###**^ Significant at *P* < 0.001.**,*** Indicate comparisons with respect to the control group. ^###^ Indicate comparisons with respect to the HFD group. Cont: Control, TQ: Thymoquinone, HFD: High-fat diet, HFD + TQ: High-fat diet + Thymoquinone.

After 12 weeks of daily HFD feeding, rats had significantly (P < 0.001) low activity of SOD, CAT, and OH-1 and expression of Nrf2 in the brains of HFD-fed rats. Additionally, the HFD-treated rats showed a significant (P < 0.001) increase in the lipid peroxidation and protein oxidation products (4-HNE and PC) as compared to the control group (Fig. 5). In contrast, TQ treatment of the HFD group significantly (P < 0.001) improved the activity of SOD, CAT, and HO-1 in the brain homogenates and the expression of Nrf2 in the pyramidal cell layer with a concomitant decrease in 4-HNE and PC in the brain when compared to the HFD group.

### TQ controlled inflammatory mediators in HFD-fed rats

**Fig. 6:**
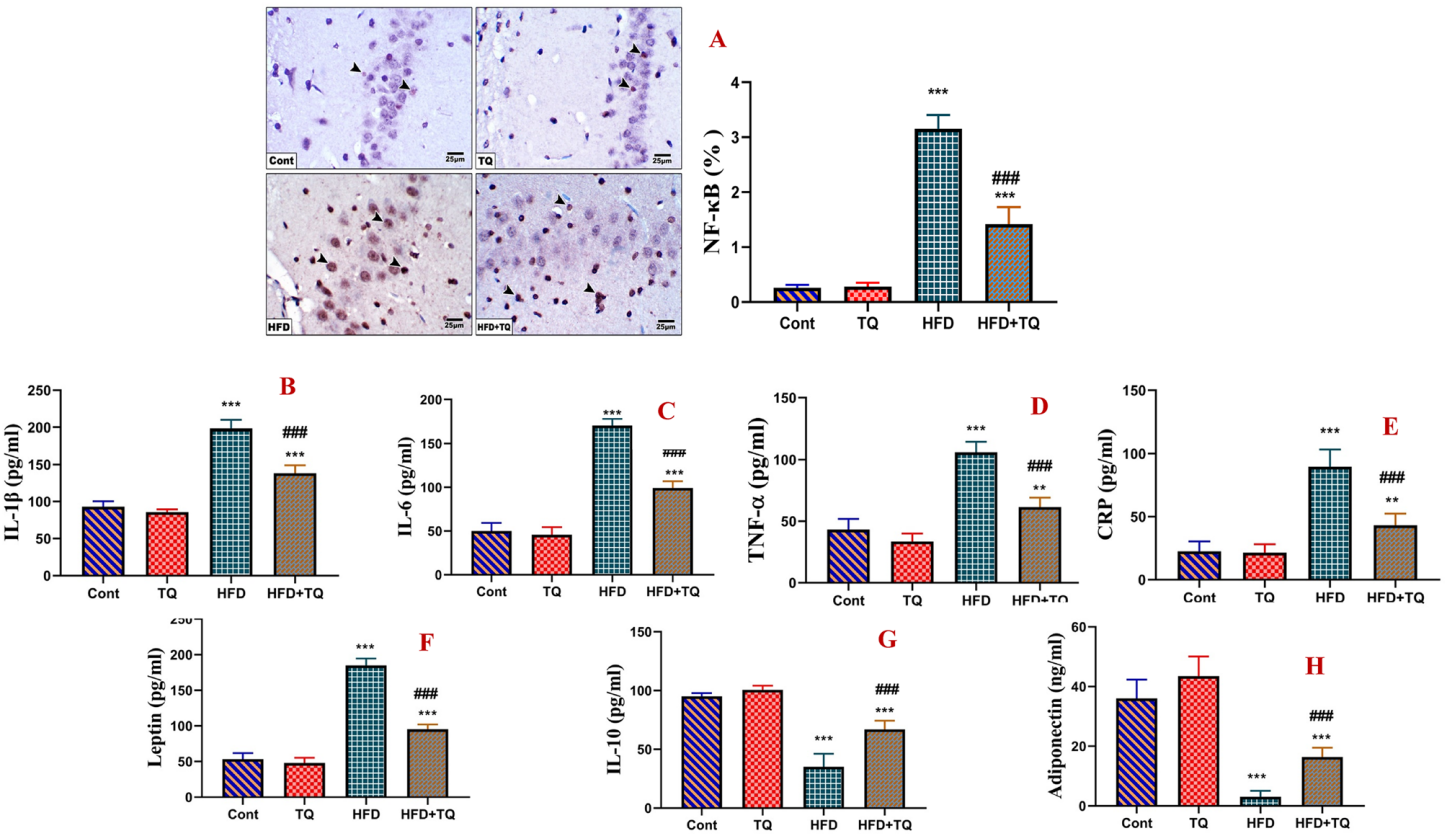
Immunohistochemical determination of the expression of NF-κB in the pyramidal cell layer of hippocampal sections in the control and different rat groups (**A**). Expression is indicated by arrowhead (IHC, 400). Values are expressed as the means ± SEM of 5 microscopic fields/tissue samples of NF-κB%. Effect of TQ and HFD on serum levels of IL-1β (pg/ml; **B**), IL-6 (pg/ml; **C**), TNF-α (pg/ml; **D**), CRP (pg/ml; **E**), leptin (pg/ml; **F**), IL-10 (pg/ml; **G**), and adiponectin (ng/ml; **H**) in all experimental groups. Data are shown as mean ± SEM (n = 6).**Significant at *P* < 0.01, and ***,^**###**^ Significant at *P* < 0.001.**,*** Indicate comparisons with respect to the control group. ^###^ Indicate comparisons with respect to the HFD group. Cont: Control, TQ: Thymoquinone, HFD: High-fat diet, HFD + TQ: High-fat diet + Thymoquinone.

Because inflammatory mediators play a remarkable role in brain diseases, inflammatory cytokines were assayed in serum (Fig. 6). HFD-treated rats showed dramatic (P < 0.001) increased levels of IL-1β, IL-6, TNF-α, leptin, and CRP levels, as well as NF-κB expression in the pyramidal cell layer, while IL-10 and adiponectin levels were decreased in the serum as compared to the control group. In contrast, HFD rats treated with TQ exhibited a significant (P < 0.001) decrease in the levels of IL-1β, IL-6, TNF-α, leptin, and CRP levels, as well as NF-κB expression in the pyramidal cell layer, with a concomitant (P < 0.001) increase in IL-10 and adiponectin when compared to the HFD group.

### TQ reduced the levels of tau and Aβ proteins in HFD-fed rats

**Fig. 7:**
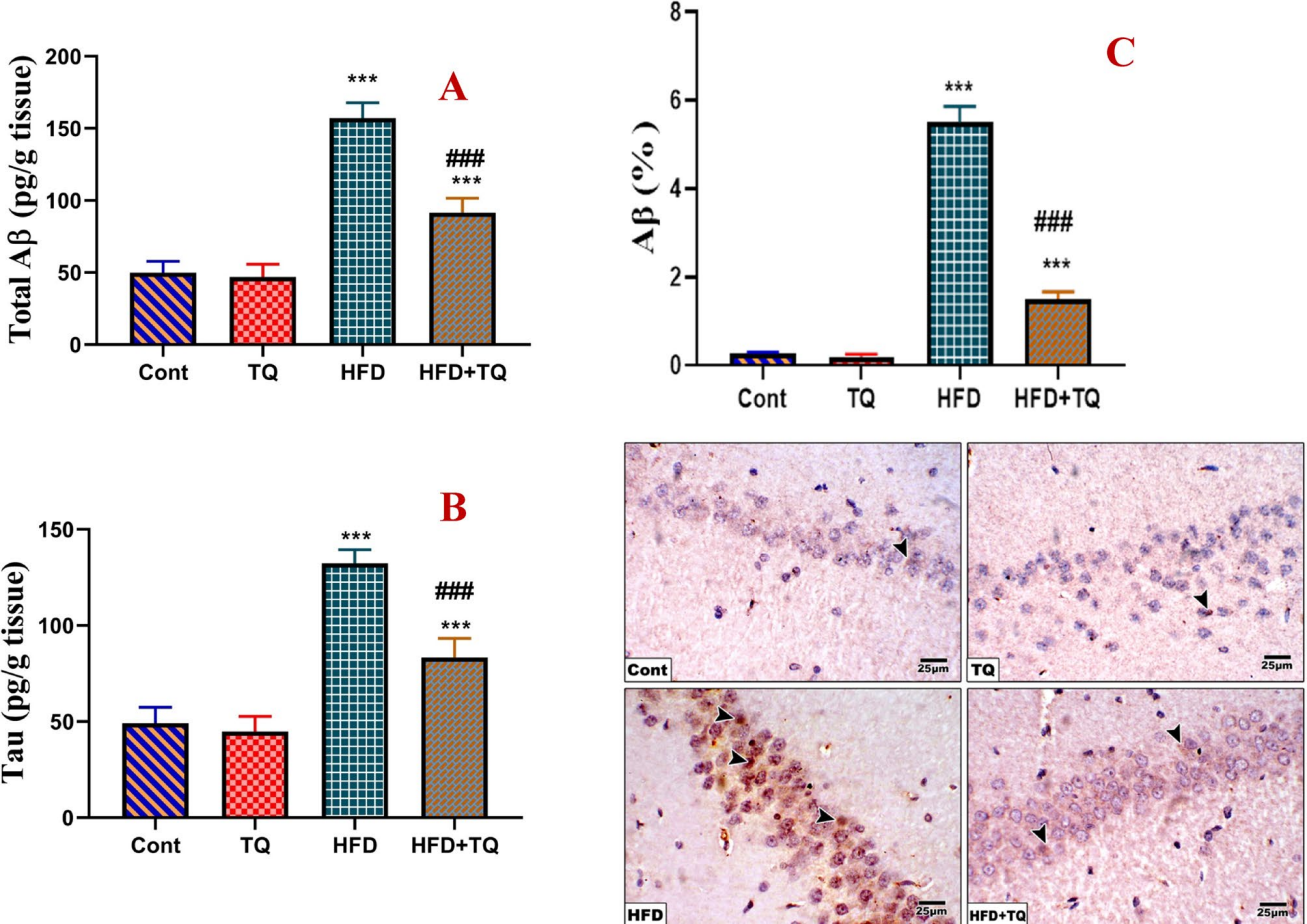
Effect of TQ and HFD on brain levels of total amyloid β (Aβ) (pg/g tissue; A) and tau protein (pg/g tissue; B) in the brain of the experimental group. Data are shown as mean ± SEM (n = 6). Immunohistochemical determination of the expression of Aβ in the pyramidal cell layer of hippocampal sections in the control and different rat groups (C). Expression is indicated by arrowhead (IHC, 400). Values are expressed as the means ± SEM of 5 microscopic fields/tissue samples of Aβ%.***,^**###**^ Significant at *P* < 0.001.*** Indicate comparisons with respect to the control group. ^###^ Indicate comparisons with respect to the HFD group. Cont: Control, TQ: Thymoquinone, HFD: High-fat diet, HFD + TQ: High-fat diet + Thymoquinone.

The current study demonstrated that HFD-fed rats had a significant (P < 0.001) increase in tau protein and Aβ levels (Fig. 7). The immunohistochemistry identification showed a significant increase in Aβ expression in the pyramidal cell layer. On the other hand, oral treatment with TQ of HFD-fed rats significantly (P < 0.001) decreased tau protein and total Aβ expression when compared with the HFD-fed rats.

### TQ interacts with the Aβ and tau protein receptor’s binding site

**Fig. 8.**
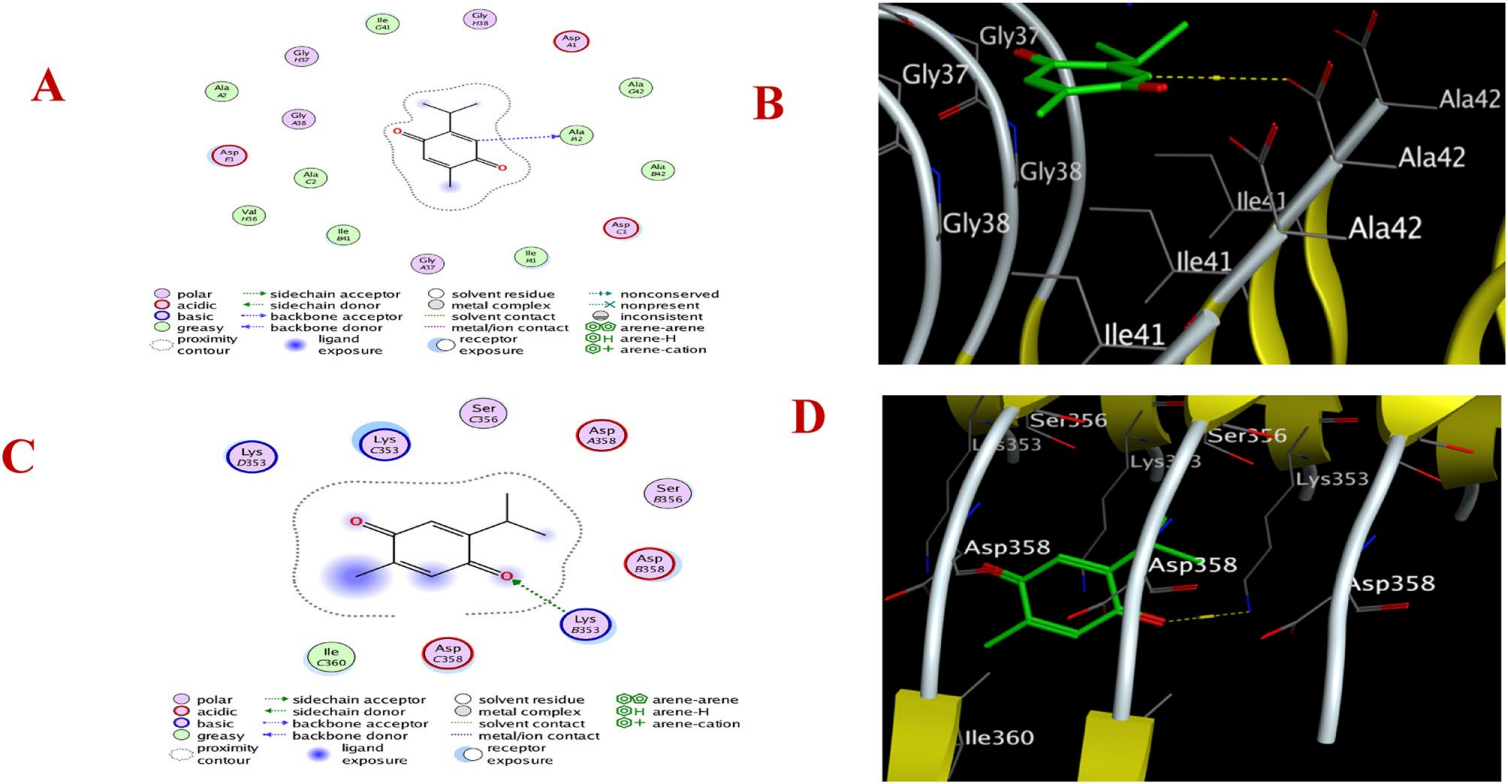
2D schematic view of TQ into the active site of the amyloid-beta receptor (A), 3D view of TQ into the active site of the amyloid-beta receptor (B), 2D schematic view of TQ into the active site of tau protein receptor (C), and 3D view of TQ into the active site of tau protein receptor (D).

In this study, molecular docking was used to predict how TQ interacts with macromolecular targets in structure-based drug design. The molecular docking simulations were carried out between TQ and the binding pockets of the Aβ and tau protein receptors to evaluate affinity scores and interaction modes (hydrophilic and hydrophobic) (Table 1). TQ was found to interact with the Aβ and tau protein receptor’s binding site, with the 2D and 3D binding characteristics (Fig. 8). The results revealed that the amino acid residue (AlaI42) of the Aβ receptor was bound through hydrogen bond to TQ. In addition, amino acid residue (LysB353) of the tau protein receptor bound through hydrogen bond to the carbonyl (C = O) group of TQ.

**Table 1:** Docking interaction of TQ with Aβ and tau proteins.

	**Protein data bank**	**Score (kcal/mol)**	**RMSD (Å)**	**Hydrophilic** **Interactions**	**Hydrophobic** **Interactions**
**Thymoquinone**	5OQV	-5.1	0.7	AlaI42	IleG41, AlaG42, AlaB42, IleI41, IleB41, AlaC2, ValH36, AlaA2
	5O3O	-4.6	1.9	LysB353	IleC360

### TQ modulated the levels of neurotransmitters and glutamine synthetase in HFD-fed rats

**Fig. 9:**
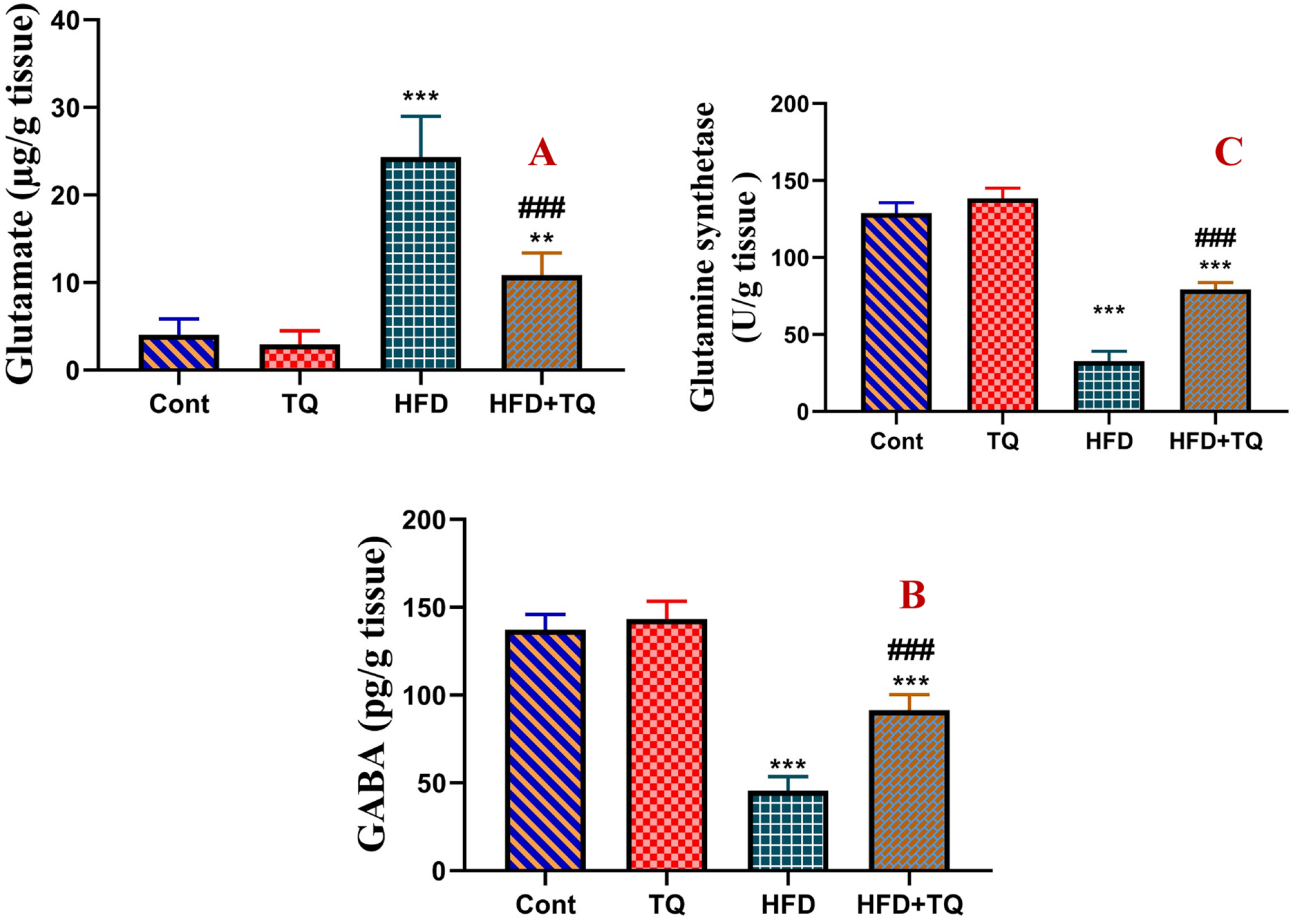
Effect of TQ and HFD on glutamate (µg/g tissue; A), GABA (pg/g tissue; B), and glutamine synthetase (U/g tissue; C) in the brain of all experimental groups. Data are shown as mean ± SEM (n = 6). **Significant at *P* < 0.01, and ***, ^**###**^ Significant at *P* < 0.001. **, *** Indicate comparisons with respect to the control group. ^###^ Indicate comparisons with respect to the HFD group. Cont: Control, TQ: Thymoquinone, HFD: High-fat diet, HFD + TQ: High-fat diet + Thymoquinone.

The levels of glutamate and GABA and the activity of glutamine synthetase in the brain tissue were displayed in Fig. 9. Daily feeding HFD for 12 weeks significantly (P < 0.001) increased the levels of glutamate, whereas GABA level and glutamine synthetase activity were significantly (P < 0.001) decreased, as compared to the control group. Additionally, a significant decrease (P < 0.001) in glutamate levels and glutamate synthetase activity and a significant (P < 0.001) increase in GABA levels were observed as compared with HFD-treated rats.

### TQ mitigated the levels of apoptosis-regulating proteins and DNA damage in HFD-fed rats

**Fig. 10:**
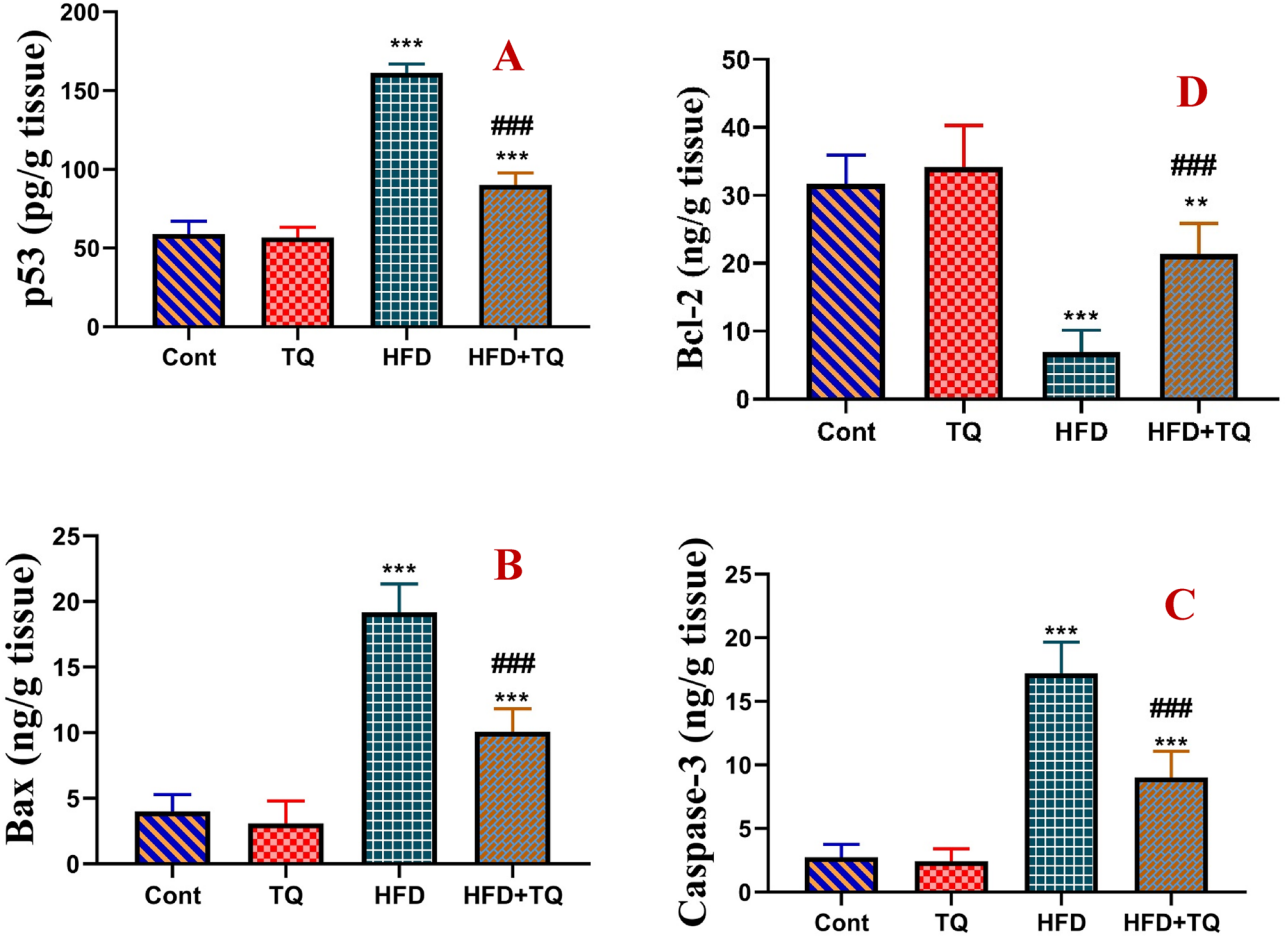
Effect of TQ and HFD on the levels of p53 (pg/g tissue; A), Bax (ng/g tissue; B), caspase 3 (ng/g tissue; C), and Bcl-2 (ng/g tissue; D) in the brains of all experimental groups. Data are shown as mean ± SEM (n = 6). **Significant at *P* < 0.01, and ***, ^**###**^ Significant at *P* < 0.001. **, *** Indicate comparisons with respect to the control group. ^###^ Indicate comparisons with respect to the HFD group. Cont: Control, TQ: Thymoquinone, HFD: High-fat diet, HFD + TQ: High-fat diet + Thymoquinone.

The impact of TQ administration on the apoptosis-regulating proteins in the brain tissue of HFD-treated rats was investigated (Fig. 10). Compared to the control group, the levels of p53, Bax, and caspase-3 increased significantly (P < 0.001), whereas the level of the antiapoptotic protein Bcl-2 was significantly (P < 0.001) reduced in the brains of the HFD-treated group. Furthermore, TQ supplementation attenuated (P < 0.001) the elevation in the levels of p53, Bax, and Caspase-3 and improved (P < 0.001) the levels of Bcl-2 in HFD + TQ in the brains of obese rats when compared to the HFD-fed rats.

**Fig. 11:**
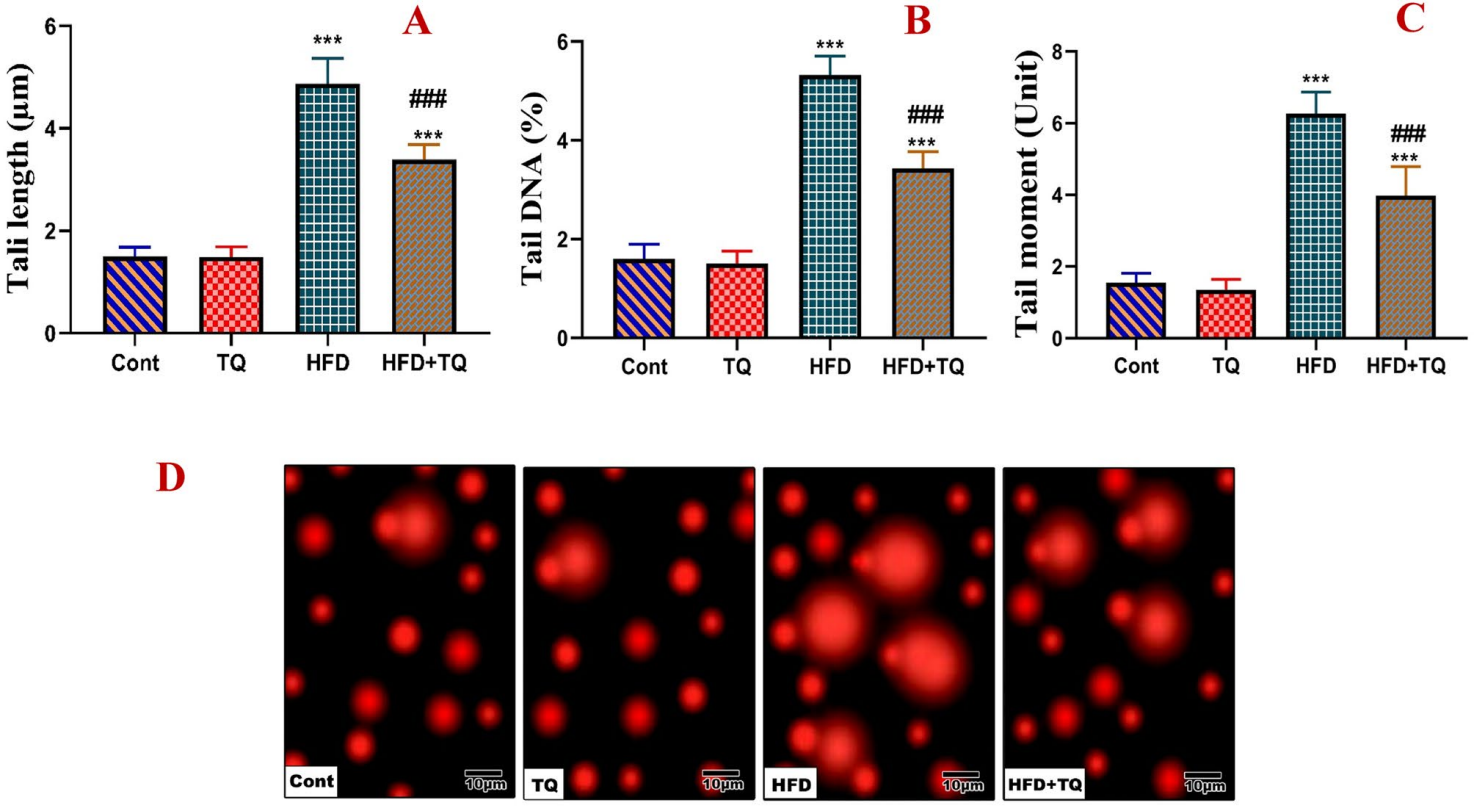
Effect of TQ and HFD on DNA damage in the brain of different experimental groups by using comet assay represented by tail length (μm; A), tail DNA (%; B), tail moment (unit; C) and (D) Representative photomicrographs of comet assay showing the effect of TQ and HFD on DNA damage.***,^**###**^ Significant at *P* < 0.001.*** Indicate comparisons with respect to the control group. ^###^ Indicate comparisons with respect to the HFD group. Cont: Control, TQ: Thymoquinone, HFD: High fat diet, HFD + TQ: High fat diet + Thymoquinone.

The ameliorative effect of TQ against DNA damage in rats suffering from obesity was illustrated using the comet assay in Fig. 11. Control and TQ-treated animals exhibited identical spots with untailed round shapes. On the other hand, all comet parameters were increased in rats given a HFD. In contrast, TQ-treated HFD-fed rats displayed considerably less DNA damage.

### TQ mitigated the histopathological changes in the hippocampus of HFD-fed rats

Histological examination of control and TQ hippocampal sections showed typical and identical arrangement of Cornu Ammonis (**CA**, **CA2**, **CA3,** and **CA4**) of the hippocampus that projects into the concavity of the dentate gyrus. CA1 and CA3 regions showed normal organization of the polymorphic layer, molecular layer, and pyramidal cell layer in between. Eosinophilic neuropil consists of the processes of neurons and glial cells and blood vessels in both molecular and polymorphic layers. In addition, the normal cytoarchitecture of the dentate gyrus was also seen.

**Fig. 12:**
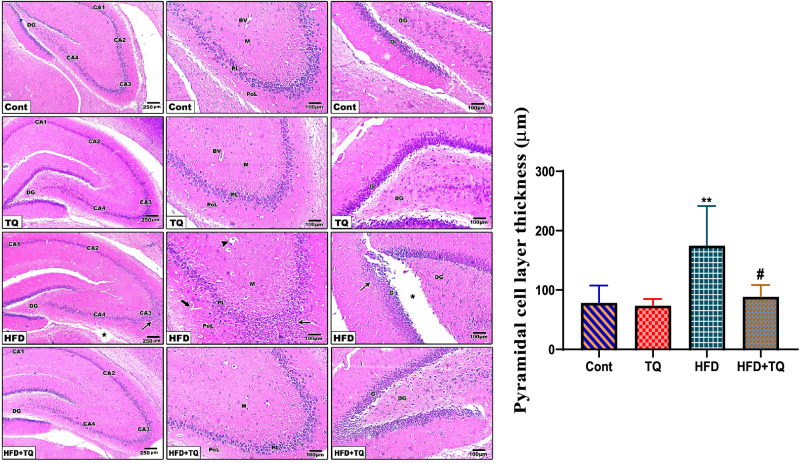
Histopathological changes in the rat’s hippocampus of the control and different treatment groups. Hippocampus section of the control and TQ-treated rats showing organized Cornu Ammonis (**CA1**), (**CA2**), (**CA3**), and (**CA4**) regions; normal dentate gyrus (**DG**) with normal polymorphic layer (**PoL**), molecular layer(**M**), and pyramidal cell layer in between (**PL**); in addition to blood vessels (**BV**). HFD-treated hippocampal sections displaying neural degeneration represented by cellular damage and separations (**asterisk**). CA1 and CA3 regions, exhibiting irregular pyramidal cell layer (**thin arrow**) with disorganized rows surrounded by pericellular halos marking neurons apoptosis mostly in molecular layer (**arrowhead**) and congestion of irregular blood vessels (**thick arrow**). Hippocampal sections of HFD + TQ rats revealing restoration of neuronal cellular organization. (H&E, X40,100). Quantification is expressed as the pyramidal layer thickness (µm) in all studied groups. Each value represents the mean ± SEM of 5 microscopic fields/tissue samples. ^#^ Significant at *P* < 0.05 and **P < 0.01respectively.** Indicate comparisons with respect to the control group.^#^ Indicate comparisons with respect to the HFD group. Cont: Control, TQ: Thymoquinone, HFD: High-fat diet, HFD + TQ: High-fat diet + Thymoquinone.

Hippocampal sections of HFD-treated rats showed neural degeneration represented by cellular damage, layer disorganization, and separations. In the CA1 and CA3 regions, the pyramidal cell layer exhibited disorganized rows with pericellular halos marking neuron apoptosis, mostly in the molecular layer, and congestion of irregular blood vessels. The thickness of the pyramidal cell layer was significantly (P < 0.01) increased with increased separation between them compared to the control group with increased granular layer separation in the dentate gyrus. Hippocampal sections of HFD + TQ rats showed restoration of cellular organization, thickness of the pyramidal cell layer, and the dentate gyrus with normal granular cell morphology and organization (Fig. 12).

## Discussion

The western diet, which consists of high-fat and refined carbohydrates, is being consumed by populations worldwide at an accelerating rate. Such diets induce obesity and are shown to be intimately associated with inflammation, oxidative stress, and neurological dysfunction^22^, including brain injury and cognitive and memory impairment in individuals of all ages^23^. Additionally, long-term high-calorie diet consumption impairs hippocampal-dependent learning and memory and alters brain plasticity^24^. TQ is a naturally occurring plant-derived product with various pharmacological and biological activities, including antioxidant and anti-inflammatory effects, among others. Nevertheless, it remains unknown whether TQ mitigates cognitive and memory performance and brain injury and the underlying mechanisms in the obese rats. The current study showed that daily TQ supplementation to the obese rats significantly improved brain plasticity and enhanced cognitive and memory performance and anxiety-related behavior through modulation of oxidative stress, inflammation, apoptosis, DNA injury and metabolic disorder.

In the present study, the effect of TQ on body weight gain of the HFD-fed rats was investigated. The current finding shows that daily administration of TQ for four weeks considerably alleviated the increase in body weight and concurrently reduced adipocyte size of visceral fats compared with the HFD-fed rats. In line with this, the TQ-treated rats showed lower serum lipid fractions, fasting glucose levels, and improved insulin resistance than the obese rats. These effects might be due to the ability of TQ to regulate lipid metabolism and gluconeogenesis in hepatic tissue and reduce intestinal glucose absorption^25^. These findings are consistent with recent data showing the anti-hyperglycemic and insulinotropic activity of TQ in rats^26^. Furthermore, TQ might have an ameliorating effect on lipotoxicity induced by HFD in rats because it promotes arylesterase activity, which protects lipoproteins from oxidation and ameliorates metabolic syndrome^27^. The decrease in adipocyte hypertrophy as well as body weight might be attributed to several mechanisms, including reduction of leptin released (as shown in the present study), appetite suppression^28^, and an inhibitory effect on pancreatic lipase^29^ suggesting that TQ might have an anti-obesity impact.

Previous studies showed that increased consumption of HFD has been associated with hyperlipidemia and linked to deficits in hippocampus-dependent learning and memory in both adults and children, indicating a detrimental effect on hippocampal function^30^. Interestingly, TQ improved hyperlipidemia with marked improvement of anxiety-related behaviors and enhanced long-term memory evidenced by increasing the number of crossings to the platform and escape latency in rats treated with HFD as measured by the Morris water maze. Furthermore, our findings revealed that TQ exerted anxiolytic effects evidenced by improving the time spent in the inner zone, rearing and grooming frequency, as well as the number of crossings in the open field test. In addition, depressive-like behavior and working memory were mitigated in HFD-fed animals treated with TQ, as indicated by the decreased immobility time and the increased spontaneous alternation percentage in forced swimming time and Y-maze test, respectively. These findings indicate that TQ counteracted memory and cognitive impairment in obese rats.

Furthermore, amyloid-β (Aβ) and tau proteins interact synergistically and serve as predictors of change in memory and spatial cognition^31^. The levels of Aβ and tau protein were assayed to evaluate the effects of TQ on the accumulation of these proteins in brains of the HFD-fed rats. The current results showed that consuming HFD significantly increased the levels of Aβ in brain homogenate and its expression in the pyramidal cell layer of the hippocampus, as well as tau proteins in the brains of the obese rats, and TQ treatment prevented the elevation of these proteins in the brain tissue. These findings are consistent with a report that TQ significantly reduced Aβ production and restored cognitive and memory deficits in a scopolamine-induced AD-like model^32^. Furthermore, TQ enhanced Aβ clearance by activation of autophagy in hippocampal and cortical neurons^33^. Recent findings showed that TQ improved synaptic recycling to mitigate Aβ clearance and inhibit Aβ aggregation^34^. These effects are supported by the current study’s molecular docking findings. TQ displayed an affinity to interact with and block the binding sites of Aβ and tau proteins, which might explain the neuroprotective effects of TQ and might be a promising supplementation for brain dysfunction.

Glutamate is the main excitatory neurotransmitter in the brain and has a role in neurophysiological functions, including learning and memory^35^. Increased glutamate levels induce neuronal excitotoxicity and cause neuronal damage with functional impairment in a number of neurological diseases^36^. Since elevated HFD consumption has been associated with impairments in recognition memory, changes in excitatory and inhibitory neurotransmitters in the brain have been observed following treatment of HFD-fed rats with TQ. It was found that TQ lowered the glutamate and upregulated the GABA levels, and restored the activity of glutamate synthetase in the brain of HFD-treated rats. These results might be attributed to the ability of TQ to reduce acute HFD-induced stress responses via upregulation of glutamate synthetase activity and reduce changes in N-methyl-d-aspartate (NMDA) receptor-induced plasticity. Glutamate upregulation activates the NMDA receptor that enhances the formation of amyloid plaque, leading to excitotoxicity and neuronal impairment^37^ suggesting that TQ alleviated HFD-induced hyperglutamatergic metabolism via the activation of glutamate synthetase. A previous study has demonstrated that TQ’s anti-amyloidogenic, antioxidant, and antiapoptotic properties can attenuate the consequences of glutamate-induced excitotoxicity^38^. Moreover, TQ restored the levels of glutamine synthetase and amylometers anxiolytic activity by modulating the levels of GABA-ergic pathways in cerebral cortical and hippocampal neurons^39,40^. Accordingly, the current data suggest that TQ might have a neuroprotective effect against glutamate-induced excitotoxicity linked with cognitive impairment in HFD individuals.

Pyramidal cells are the main excitatory neurons found in several areas of the brain, including the hippocampus, and are aligned in the pyramidal cell layer. Pyramidal cells in the subiculum and CA1 in the hippocampus are responsible for creating a cognitive map that encodes spatial, contextual, and affective information, which is then transmitted throughout the brain^41^. In the current study, rats fed a HFD displayed an enlarged pyramidal cell layer in the CA1 in the hippocampus, which is indicative of disrupted hippocampal structure, compared to the control rats. In contrast, obese rats treated with TQ displayed a thinner layer of pyramidal cells than obese rats, suggesting that TQ might have a protective impact on pyramidal cells in the hippocampus, hence improving cognitive performance. This finding confirms a study reporting the healing effect of TQ on the pyramidal cell layer in the CA1 after traumatic brain injury via suppression of oxidative stress^42^.

A recent study has reported that chronic consumption of HFD disrupted redox imbalance by accelerating β-oxidation of fatty acids and increasing the production of ROS^43^. According to Zhang, et al.^44^, cognitive deficits are associated with HFD-induced oxidative stress. The current results showed that daily feeding HFD for 12 weeks dramatically increased the levels of protein oxidation and the lipid peroxidation markers (4-HNE and PC, respectively) as well as a notable decrease in SOD and CAT activity in the brain. Meanwhile, TQ treatment enhanced the activity of SOD, CAT, and HO-1 levels in the brain and Nrf2 expression in the pyramidal cell of obese rats. Moreover, the reduction in PC and 4-HNE levels indicated the alleviation of oxidative stress via activation of the antioxidant system. These findings agree with a study showing that TQ increased the expression of Nrf2 and HO1 and SOD activity in the hippocampus^45^. A recent study showed that TQ ameliorated oxidative damage in dopaminergic neurons by activating the Nrf2/ARE pathway, reducing lipid peroxidation, and increasing the expression of HO-1, thus mitigating the propagation of neural damage^46^. Because TQ has been shown to exhibit antioxidant effects based on its chemical structure^13^, targeting protein and lipid oxidation by TQ might counteract the damage caused by oxidative stress.

Obesity and overweight are linked to chronic systemic inflammation, which can accelerate the progression of neurodegenerative diseases. The current study displayed a marked decrease in the levels of IL-1β, IL-6, CRP, TNF-α, and leptin with a significant increase in adiponectin and IL-10 in the sera of HFD-fed rats treated with TQ, suggesting amelioration of the unbalanced cytokines. These results agreed with studies that reported that TQ reduced IL-6, TNF-α, IL-1β, CRP, and TNF-α in the rat hippocampus^47,48^. Additionally, TQ downregulated NF-κB expression in the pyramidal cells in the hippocampus of HFD-fed rats. This effect matches a recent study that TQ has immunomodulator activities through controlling NF-κB and Toll-like receptors, therefore regulating the expression of pro-inflammatory mediators^49,50^. The increase of adiponectin in HFD rats treated with TQ showed beneficial effect. It can enhance insulin sensitivity, stimulate fatty acid oxidation, and reduce gluconeogenesis in the liver^51^. Moreover, it exhibits an anti-inflammatory effect by downregulating NF-κB and TNF-α-induced expression of endothelial adhesion molecules and enhances IL-10 expression in the brain^52,53^.

In addition to regulating energy balance and glucose and lipid metabolism, leptin and adiponectin, which are mostly produced by adipocytes, also modulate inflammation and may be implicated in the link between obesity and neuronal function^54^. The current study demonstrated that TQ administration improved the balance between leptin and adiponectin levels in the obese rats. This result confirms other studies that rats given a 50% HFD showed a reduction in leptin and lipid blood levels after receiving TQ treatment for 6 weeks^55^. This effect might be explained by the reduction of adipocyte size, body weight, and pro-inflammatory cytokines, suggesting that TQ has an anti-inflammatory effect.

Neuronal apoptosis is associated with HFD-induced brain damage^56^. Previous findings revealed that HFD-fed rats had higher oxidative stress in the brain and hippocampal regions, leading to impaired hippocampal plasticity and increased apoptosis^57^. Assessing apoptotic-regulating proteins in the brains of obese rats allowed further mechanistic pathways of TQ’s neuroprotective effects and the improved cognitive function in HFD-fed rats. In line with Tabeshpour, et al.^58^, the current findings demonstrated that TQ had an antiapoptotic effect by reducing Bax, caspase-3, and p53 while upregulating Bcl-2. These data revealed the ability of TQ to counteract the mitochondrial pathway of apoptosis and maintain mitochondrial integrity against HFD-induced apoptotic response. It is reported that TQ decreased the expression of the caspase-3 in the brain tissue^15,59^. TQ protected neuronal viability against arsenic-induced hippocampal damage by improving mitochondrial membrane potential, decreasing mitochondrial dysfunction, restoring the levels of Bcl-2 and Bax through improving redox balance^60,61^. Moreover, TQ protected human neuronal cells (SH-SY5Y cell line) by modulating several signaling genes p53, AKT1, ERK1/2, P38 MAPK, JNK, and NF-κB^62^.Thus, the antiapoptotic effects of TQ afford neuroprotection against HFD-induced brain injury.

Numerous neurological disorders are linked to the loss or deterioration of genome repair processes, which can especially affect the DNA of neurons. It is commonly known that the neuronal genome experiences DNA damage, most likely due to high levels of oxidative stress in the brain and the high rate of oxygen consumption in the brain and the corresponding generation of ROS in the brain^63^. Oxidative stress associated with obesity is strongly contributed to the progression of DNA damage^64^. Our study revealed that TQ treatment markedly protected against DNA damage in obese rats, as indicated by low comet parameters compared with the brains of HFD-fed rats. These results suggest that TQ protected DNA structure and enhanced DNA repair mechanisms, possibly through the base excision repair (BER) pathway^65^. The improvement may also be due to TQ’s capacity to modulate redox imbalance and strengthen the neuronal antioxidant system^66^. TQ increased the expression of Nrf2, which is a master regulator of antioxidant signaling, to reduce ROS-induced DNA fragmentation^67^. The current findings showed that TQ is effective in maintaining DNA integrity caused by consuming HFD.

## Conclusions

**Fig. 13:**
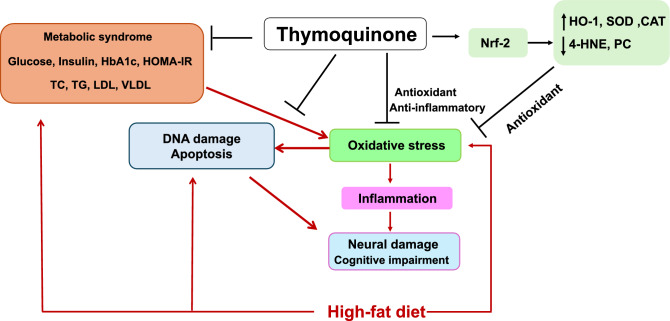
Graphical illustration of the several mechanisms through which thymoquinone reduced obesity and improved memory and cognitive function in rats fed a high-fat diet.

In conclusion, the current findings suggest that TQ maintains brain plasticity and cognitive and memory function through several interconnected pathways. These include reduction of the oxidative stress and metabolic disruption via its antioxidant properties acquired from its chemical structure and by enhancing antioxidant capacity via the Nrf2/OH-1/SOD/CAT pathway. The TQ-induced improvement of the redox balance modulated neuroinflammation and DNA damage, which moderated the mitochondrial apoptosis pathway, hence protecting neurons and their functions. These effects help regulate neurotransmitter balance and down-regulate the accumulation of Aβ and tau proteins, which results in protection of brain injury and maintenance of neuronal cognitive and memory function (Fig. 13). Consequently, TQ may be recommended as a supplement for obese individuals to maintain brain health and cognitive fitness. However, further research is required to fully understand the complex interaction between Nrf2 expression and brain challenges, taking into account different neurodegenerative diseases associated with obesity.

## Materials and methods

### Chemicals

Thymoquinone (TQ) was purchased from Sigma Chemical Company (Sigma Aldrich, St. Louis, MO, USA) (CAS number 490–91-5). All other chemicals used in this study were of the highest chemical grade.

### Animals

Twenty-four adult male Wistar albino rats weighing 120–140 g were obtained from the Egyptian Institute of Vaccine and Serological Production, Egypt. Rats were maintained at the animal house of the zoology department, Faculty of Science, Mansoura University. The rats were housed in a polypropylene cage with a 125 ml water bottle. Bedding made of straw pellets was used and cleaned every 2 days. All rats were acclimatized to the standard laboratory conditions (25 °C and a 12-h light/12-h dark cycle) and had free access to standard commercial rat chow and water ad libitum. The methods are approved by the ethical research project committee in Mansoura University (MU-ACUC.SC. PhD.24.01.16) and performed in accordance with its relevant guidelines and regulations complied with ARRIVE in accordance with Guidance on the operation of the Animals, Act 1986 and associated guidelines.

Rats are frequently utilized in behavioral studies because they are more gregarious than mice and their behavior more closely resembles that of humans. Furthermore, rats exhibit less stress when handled by humans. The pharmaceutical sector heavily relies on rats for toxicity^68^.

### Experimental design and animal treatment

**Fig. 14:**
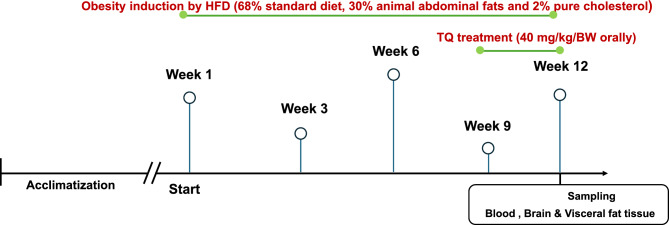
Schematic illustration of the experimental design. Four groups (n = 6) of rats were randomly assigned after two weeks of acclimatization. The control group received a standard diet. Rats in the thymoquinone (TQ) group were daily administered TQ orally daily from week 9 to week 12. Rats in the high-fat diet (HFD) group were given HFD-fed pellets for 12 weeks. Rats in the HFD + TQ-treated group received HFD daily for 12 weeks and were treated with TQ daily from week 9 to week 12.

After two weeks of acclimatization, a total of 24 animals were randomly divided into four groups of 6 rats each. The control group received a standard diet (29% calories from protein, 54% from carbohydrates (no sweetener added), and 17% from fat). Rats in the thymoquinone (TQ) group were administered TQ orally by stomach tube daily from week 9 to week 12 at a dose of 40 mg/kg body weight^69,70^. Rats in the high-fat diet (HFD) group were given HFD-fed pellets (68% standard diet, 30% animal abdominal fat, and 2% pure cholesterol) for 12 weeks^71^. The HFD was available ad-libitum. Rats in the HFD + TQ-treated group received HFD daily for 12 weeks and were treated with TQ (40 mg/kg body weight) daily from week 9 to week 12 in similar way of groups 2 and 3 (Fig. 14).

Body weights were measured weekly starting at the first day of the experiment for 12 weeks.

### Sample collection

Rats were fasted overnight and then anaesthetized with a mixture of ketamine and xylazine (1 ml/kg BW, IP). Blood was obtained by cardiac puncture into a non-heparinized tube, and clear serum was separated after clotting and cool centrifugation. The clear non-hemolyzed sera were stored at -20 °C until biochemical analysis. A small amount of blood was collected in an EDTA tube for glycosylated hemoglobin (HbA1c) determination. Furthermore, brain and visceral fat tissue was immediately separated and fixed in neutral formalin (10%) for histopathological investigation. Other samples of brain were removed, weighted, and homogenized (10% w/v) in cold tris–HCl buffer (0.1 M) pH 7.4. The homogenates were cool centrifuged for 15 min at 4000 rpm. The supernatant was properly aspirated, divided into aliquots, and stored at—20 °C until biochemical analysis.

### Neurobehavioral tests

Exploratory, long-term spatial memory, depression, and short-term spatial memory were assessed by the open field test, Morris water maze test, forced swimming test, and Y-maze test, respectively. All tests were performed in a sound-proof room.

### Open field test

The exploratory, locomotor, and anxiety-related behaviors were evaluated by the open field test. The experimental animal was placed in a black square box measuring 40 cm (W) × 40 cm (L) × 30 cm (H). The square box was divided into 16 equally sized squares. Each experimental rat was placed in the center of the box and allowed to explore the square box for 10 min freely. At the end of each trial, the square box was cleaned with 70% ethanol. Time spent in the inner zone, number of line crossings, rearing frequency, and grooming frequency were evaluated as previously described^72,73^.

### Morris water maze test

To evaluate the effect of HFD on long-term spatial memory and learning, rats were subjected to the Morris water maze. A circular tank (150 cm diameter × 50 cm height) was filled with water and provided with a white platform (10 cm diameter). The tank was divided into four equal quadrants, marked N-S-E-W. In addition, the platform was placed in the middle of one of the four maze quadrants (the target quadrant) and submerged 1.5 cm below the water level. Rats were trained to find the platform in three trials per day for three days. Each trial begins with a different starting position (in a quadrant not containing the platform). Rats were allowed to swim freely for 60 s to find the platform, then allowed to stay on it for 20 s before being returned to their home cage and dried with a pad until the next trial. If the rat had failed to reach the direct platform, the rat was directed to the platform and given 20 s to remain there. On the 4^th^ day, after all 9 trials were completed, a probe test was performed to confirm the animal’s behavior and determine the position of the platform. Additionally, escape latency and the number of the platform location crossings were evaluated.

### Forced swimming test

The forced swimming test was used to evaluate depression-related behaviors. Each rat was permitted to swim for 6 min in a transparent cylindrical tank (40 cm height × 20 cm diameter) filled with water to a depth of 25 cm to prevent the rat from reaching the bottom of the container with its hind limbs or tail. Immobility time was recorded in the last 4 min, while the initial two minutes were referred to as habituation time. After that, the rat was dried with a pad and returned to their plastic cage^74^.

### Y‑maze test

The Y-maze test was used to evaluate the effect of HFD on short-term working memory by measuring the percentage of spontaneous alternation behavior. Y-maze consisted of three equal arms labeled A, B, and C, respectively. Additionally, each arm is 50 cm long, 20 cm high, and 15 cm wide. Each rat was placed at the end of one arm and permitted to explore the maze freely for 8 min. A rat was considered to be in one alternation when it entered each of three successive arms. The following formula was used to estimate alteration percentage; number of spontaneous alternations)/ (total number of arm entries − 2) × 100^75^.

### Biochemical analysis

Quantitative determination of serum glucose was performed calorimetrically using kits provided by Spinreact (Spain). Insulin levels in serum were determined according to the instructions of the ELISA kits (**Catalog #** MX41011 and ELR-Insulin) RayBiotech (Georgia, USA). Homeostatic model assessment of insulin resistance (HOMA-IR) was calculated according to Majid, et al.^76^. The levels of HbA1c were measured calorimetrically by a commercial kit (**Catalog #** 80,300) obtained from Crystal Chem (Elk Grove Village, USA).

Serum lipid fractions (total cholesterol (TC), triglyceride (TG), and high-density lipoprotein cholesterol (HDL-C)) were estimated using enzymatic colorimetric assay kits (**Catalog #** SPIN-1001090, SPIN-1001310, and SPIN- 1,001,095) purchased from Spinreact (Girona, Spain), respectively. The levels of LDL-C and V-LDL-C were calculated according to Friedewald, et al.^77^.

Serum levels of interleukin-1 beta (IL-1β), interleukin-6** (**IL-6), tumor necrosis factor alpha (TNF-α), interleukin-10 (IL-10), and C-reactive protein (CRP) were determined quantitatively using rat ELISA kit (**Catalog #** ELR-IL1b, ELR-IL6, ELR-TNFa, ELR-IL10, MBS2508830) purchased from RayBiotech (Georgia, USA) and MyBioSource (San Diego, CA, USA), respectively. Quantitative determination of leptin and adiponectin was determined in serum using a double antibody sandwich ELISA kit (**Catalog #** ELR-Leptin and ELR-Adiponectin) purchased from RayBiotech (Georgia, USA).

Quantitative determination of 4-Hydroxynonenal (4-HNE) and protein carbonyl (PC) in brain homogenates were determined using rat ELISA kits (**Catalog #** MBS7606779 and MBS2600784) provided by MyBioSource (San Diego, CA, USA). In brain homogenates, the activity of superoxide dismutase (SOD) and catalase was determined by colorimetric assay kits (**Catalog #** SD 25 21 and CA 25 17) purchased from Biodiagnostic Company (Giza, Egypt). The concentration of heme oxygenase 1 (HO-1) was determined quantitatively using rat ELISA kit (**Catalog #** MBS764989) purchased from MyBioSource (San Diego, CA, USA).

The levels of total amyloid beta peptide (Aβ) concentration and tau protein in the brain homogenates were performed quantitatively using rat ELISA kits (**Catalog #** CEA946Ra and MBS3807667) purchased from Cloud-Clone Corp (Wuhan, China) and MyBioSource (San Diego, California, USA), respectively. Glutamate, gamma-aminobutyric acid (GABA), and glutamate synthetase in brain homogenates were assayed quantitatively using rat ELISA kits (**Catalog #** KA1909, MBS269152, and MBS764194) obtained from Abnova (Taipei City, Taiwan) and MyBioSource (San Diego, CA, USA), respectively.

The levels of Bax, Bcl-2, p53, and Caspase-3 were determined quantitatively using the instructions of the ELISA kits (**Catalog #** MBS2512405 and MBS2515143, CSB-E08336r, and CSB-E08857r) provided by MyBioSource (San Diego, CA, USA) and Cusabio (Wuhan, China), respectively.

### Comet assay

In brain homogenates, DNA damage was determined using single-cell gel electrophoresis (comet assay) method^78^. The Komet 5.0 image analysis software was used to determine the length of DNA migration (μm), the percentage of migrated DNA (%), and the tail moment to assess the quantitative and qualitative amount of DNA damage in the brain (Unit). Using CASP software, the tail length was multiplied by the percentage of migrated DNA to calculate the tail moment.

### Molecular docking

#### Preparation of Amyloid beta-42 and tau protein

Molecular docking investigations are conducted to understand the binding interactions and specific amino acid residues responsible for the biological activity of thymoquinone. The fibril structure of Aβ is available under PDB ID: 5OQV^79^, while the fibril structure of tau protein can be found under PDB ID: 5O3O^80^. Aβ and tau protein receptors were obtained from the protein data bank (https://www.rcsb.org/). Receptor protein was prepared for molecular docking by 3D protonation, hydrogen addition, energy minimization, and prediction of the active site for ligands. Next, thymoquinone was docked with the target proteins (Aβ and tau protein) using the Triangle Matcher placement method while using MOE software. Then, the ligand was selected, and rescoring was set at London dG and rescoring at GBVI/WSA dG, running so as to note the ligand interaction with protein^81^. Protein–ligand docking score, ligand properties, and 2D and 3D structures were assayed.

### Ligand preparation

The MOE builder tool was used to construct the 3D structure of TQ. Afterward, the 3d structure was minimized to reduce the strain within the bond and added to MOE databank.

### Histopathological examination of the hippocampus and visceral fat

For histological investigation, brain and visceral fat tissues were preserved in 10% neutral formalin (pH 7.6). After undergoing dehydration, cleaning, and paraffin infiltration, the samples were sliced into 5-µm slices. The sections were stained with hematoxylin and eosin. A bright field Olympus light microscope and Amscope MU1000 camera were used to capture images to look for histopathological alterations in the hippocampus and visceral fat.

### Immunohistochemical evaluation of Nrf2, NF-kB, and Aβ

According to Janardhan, et al.^82^, labeled streptavidin–biotin immunoperoxidase technique was used. Paraffin-embedded brain sections were deparaffinized, and antigen retrieval was done by heat-treating. Afterward, the activity of endogenous peroxidase was blocked by 3% H_2_O_2_. Following the manufacturer’s instructions, the sections were blocked with 5% bovine serum albumin and then incubated overnight at 4 °C with primary antibodies, including anti-Nrf2 rabbit polyclonal antibody (Catalog # A11159; ABclonal, Wuhan, Hubei, China; 1:200), Aβ mouse monoclonal antibody (Catalog # 803,001; BioLegend, San Diego, USA; 1:2000), and NF-kB mouse monoclonal antibody(Catalog # EM1377; ELK Biotechnology, Denver, USA; 1:200). Slides were then examined and photographed using the Olympus digital camera installed on Olympus microscope with a 1/2 X photo adapter, using 10 X objective. The result images were then analyzed for labeling index using ImageJ software as previously described^83^.

### Statistical analysis

All data values were presented as mean ± the standard error of the mean (SEM). Statistical comparisons were made by one-way analysis of variance (ANOVA) and Tukey as a post-hoc test by GraphPad prism 8.0 software. In addition, statistical differences were represented with the following symbols of significance level: *P < 0.05, **P < 0.01 and ***P < 0.001 versus control animals. ^#^P < 0.05, ^##^P < 0.01 and ^###^P < 0.001 versus HFD group.

## Data Availability

All data generated or analyzed during this study are included in this published article.
